# Advancements in Skin Delivery of Natural Bioactive Products for Wound Management: A Brief Review of Two Decades

**DOI:** 10.3390/pharmaceutics14051072

**Published:** 2022-05-17

**Authors:** Cameron Ryall, Sanjukta Duarah, Shuo Chen, Haijun Yu, Jingyuan Wen

**Affiliations:** 1 School of Pharmacy, Faculty of Medical and Health Sciences, The University of Auckland, Auckland 1010, New Zealand; crya086@aucklanduni.ac.nz (C.R.); s.duarah@auckland.ac.nz (S.D.); 2 Shanghai Institute of Materia Medica, Chinese Academy of Sciences, Shanghai 201203, China; hjyu@simm.ac.cn

**Keywords:** wound healing, natural products, advanced delivery, traditional medicine, alternative medicine

## Abstract

Application of modern delivery techniques to natural bioactive products improves their permeability, bioavailability, and therapeutic efficacy. Many natural products have desirable biological properties applicable to wound healing but are limited by their inability to cross the stratum corneum to access the wound. Over the past two decades, modern systems such as microneedles, lipid-based vesicles, hydrogels, composite dressings, and responsive formulations have been applied to natural products such as curcumin or aloe vera to improve their delivery and efficacy. This article reviews which natural products and techniques have been formulated together in the past two decades and the success of these applications for wound healing. Many cultures prefer natural-product-based traditional therapies which are often cheaper and more available than their synthetic counterparts. Improving natural products’ effect can provide novel wound-healing therapies for those who trust traditional compounds over synthetic drugs to reduce medical inequalities.

## 1. Introduction

The field of wound-healing medicine has been advanced with modern technologies and modern drugs despite traditionally used natural products’ known effectiveness. Wounds affect both developed and developing nations, yet they are generally treated differently. The healing process can be painful, develop into an infection, or come across obstacles such as bacterial biofilms causing the wound to become chronic [1,2]. As a prevalent and visible condition, multiple traditional/alternative wound-healing medicines exist from historic medical communities [3,4].

The WHO defines traditional medicines as *‘the sum total of the knowledge, skill, and practices based on the theories, beliefs, and experiences indigenous to different cultures, whether explicable or not, used in the maintenance of health as well as in the prevention, diagnosis, improvement or treatment of physical and mental illness’* [5]. In 2019, the World Health Assembly formed resolutions promoting traditional medicines, especially in primary healthcare [5]. This resolution arose from an ever-increasing interest and belief that traditional natural medicines are safer and more commonly available than conventional drugs [6]. Cultures such as China and Egypt with established traditional practices use traditional medicines the most; however, it is a worldwide phenomenon, with 60% of the world’s population estimated to rely on traditional medicine [7]. Traditional medicines are often used more in developing nations due to cultural norms or superior accessibility. Natural products already culturally established in developing nations should be re-examined as medicines to provide unique solutions to medical inequalities. Low bioavailability and poor solubility because of limited membrane permeability restricts many natural compounds from their full potential [8]. An example is the anti-inflammatory escin from horse-chestnut, which is poorly lipid-soluble and has a bioavailability of 12.5% [9]. Traditional medicine has gained popularity due to growing distrust of conventional medicine, and as traditional medicine rises in popularity, research should go into its safety, efficacy and optimisation [10]. 

This review describes how natural wound-healing products are being reapplied with modern delivery strategies. Additionally, we describe how different kinds of wounds form and heal, and then how they are treated. We categorize compounds by their bioactive function and then discuss novel delivery strategies. Our aim is to provide a new perspective on the future of natural products and the problems the field may face.

## 2. Wound-Healing Physiology

**Figure 1:**
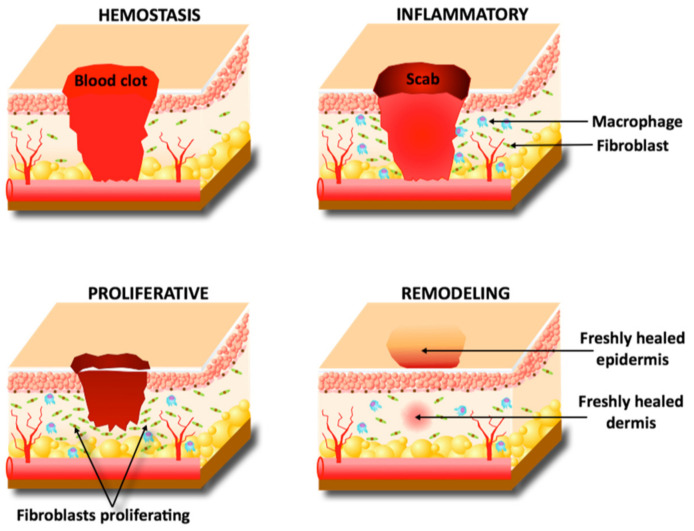
Wound self-healing process.

A wound is either acute or develops into a chronic wound due to various factors such as systemic illnesses and repeated trauma. An acute wound is defined as an injury to tissue healing within 8–12 weeks [11]. Acute wounds are commonly caused by burns, lacerations, and abrasions and have a four-stage healing process: hemostasis, inflammation, proliferation, and maturation [11,12]. The four stages are diagrammatically presented in Figure 1. Chronic wounds differ from acute wounds in their timeframe; differently to acute wounds, chronic wounds are yet to heal after 12 weeks and often reoccur [13]. Pre-existing conditions such as diabetes or repeated damage are common causes of chronic wounds and can lead to ulcers or amputations [14].

An acute injury’s first phase of healing is hemostasis. This is clotting of the blood to prevent blood loss while also creating a temporary scaffold for future cells’ use [2]. Immediately following the injury and during the hemostasis phase, cytokines recruit inflammatory cells to clear the wound area of foreign material and dead tissue [15]. With the wound now sealed and an immune response taking place, fibroblasts and keratinocytes migrate into the damaged area to begin the proliferation phase—some literature views this migration as a phase of healing itself [11]. Keratinocytes proliferate to cover the dermis and reform the epidermal barrier beneath the clot, while fibroblasts begin secreting collagen and extracellular matrix (ECM) to repair the dermis [15]. Capillary reformation responds to angiogenic growth factors such as vascular endothelial growth factor (VEGF) once the dermis has reformed enough to support vessel growth [2]. Finally, myofibroblasts contract the wound area while keratinocytes proliferate to reform its full epithelium, finishing the healing process [13].

Chronic wound healing is often stalled during the inflammation phase [2]. Prolonged inflammation causes imbalances in potent cytokines only meant to be present for a short time [16]. Such an imbalance can increase the degradation of ECM growth factors and receptors [16]. The degradation products of the breakdown require a continued immune response, beginning a cycle of non-healing and persistent inflammation [2,16]. The excess workload placed on immune cells impairs their function and allows bacteria to infiltrate the wound and form a multicellular plaque called a biofilm. This film is typical of staphylococcus aureus and other airborne bacteria; the film covers the topical surface of the wound and can block the delivery of healing pharmaceutics [1]. Breaking the cycle of inflammation and ECM breakdown and removing the biofilm layer are popular research areas aiming to treat the 6.5 million people diagnosed with chronic wounds each year [17]. 

## 3. Conventional Treatment

### 3.1. TIME and TWA: Deciding on a Treatment

A key focus in today’s conventional treatment of wounds is managing the risk of a wound becoming chronic [18]. A wound should only require treatment if its natural healing is stalled or interrupted. The ‘TIME’ or ‘TWA’ processes are used to assess the wound and determine a treatment. No single treatment is applied to all wounds; hence, assessing wounds and making evidence-based decisions is emphasised [18,19]. Both TIME and TWA are acronyms. For TIME, T stands for ‘tissue non-viable or deficient’, I for ‘infection or inflammation’, M for ‘moisture balance’, and E for ‘edge of wound—non-advancing or undermined’ [18,20]. TWA stands for ‘Triangle of Wound Assessment’ [21,22]. TWA elaborates on TIME with a three-point ‘triangle’: wound bed, wound edge, and peri-wound skin [22]. TWA and TIME analysis provide a systematic way to analyse a wound and direct clinicians to the proper treatment.

### 3.2. Wound-Healing Dressings

The most common and historically present product for a wound is its dressing. The primary function of a dressing is to protect against foreign microbes and protect the skin from exudate [21,23]. It is often desirable for a dressing to be absorbent, particularly when a wound has too much exudate interfering with the patient’s daily life or creating too friendly an environment for bacteria to colonise [20]. Absorbents can be fibrous, fabric, or a combination of the two: these forms are among the most accessible wound dressings around the world [19]. Modern technologies have added to this category with hydrofibres and impregnated gauzes, which are more bioactive than their fibre/fabric counterparts [23]. Such direct dressings can have the disadvantage of being painful when removed, hence the use of non-adherent dressings. Non-adherent dressings are often preferred for the contact layer dressing as they do not attach to the wound’s tissue. Non-adherence allows the protection of granulation tissue and the epithelium at the expense of requiring a secondary dressing to secure the wound [19,22]. 

Water-based formations such as hydrogels and hydrocolloids are applied to wounds requiring a moist environment to heal. Hydrogels use hydrophilic polymers to attract water and create a three-dimensional network in the form of a gel. Hydrogels can be used to rehydrate a wound bed, rehydrate necrotic tissue, absorb excess exudate, or reduce pain [19,23,24]. Hydrogels can be loaded with bioactive compounds to deliver it directly to the wound area in a relatively painless manner. An available hydrogel is Curasol™ which is applied to an absorbent layer such as cotton or gauze and then taped, netted, or bandaged onto the wound. Hydrogel dressings often market themselves based on their pain relief and rehydrating abilities—one problem with these products is the complicated application that requires a healthcare professional [25]. Hydrogels are commonly applied to traditional medicine and are often fabricated using polymers derived from nature [26,27,28]. The polymers applied to hydrogels are becoming increasingly advanced; Yan et al. formulated a thermosensitive hydrogel that released a phage to combat bacterial infections. This hydrogel could be infected as a liquid but would form into a hydrogel dressing once exposed to the temperature of an inflamed wound bed [29]. Hydrocolloid dressings such as alginates have the advantage of requiring no secondary dressing. Hydrocolloid dressings are gel-forming agents that absorb and retain fluid to create a wound-moistening gel [24,30]. Hydrocolloids, like hydrogels, can be loaded with antiseptics or wound-healing drugs to make them bioactive. Hydrocolloid dressings derived from sodium alginate taken from brown seaweed are commonly used for wounds with excess exudate because of their absorptive properties. Other hydrocolloids such as Duoderm™ can be used for shallow acute wounds with little exudate present [24,31]. Hydrocolloids and hydrogels reduce pain sensation by keeping nerve endings moist and providing a provisional ECM to facilitate autolysis [24,30]. 

Semipermeable dressings such as foams and films can protect wounds from the external environment while allowing essential molecules to enter. Foams fabricated from polyurethane or silicone can protect against bacteria and moisten the wound bed while avoiding tissue damage when removed [32]. Foams are also used to encapsulate drugs and have been applied with natural products such as asiaticoside from *Centella asiatica* [33]. Recent advances in foams for wound care combine other technologies to create responsive/smart dressings. A multi-layer dressing made from an anti-microbial foam and an electrospun cellulose surface mesh was formulated by He et al. which changes colour in response to infection-caused pH changes [34]. Films are like foams by being semipermeable but are more often applied to epithelizing wounds with little exudate. Oxygen and carbon dioxide can pass through films but, similarly to foams, bacteria or pathogens are kept away. Koetse et al. and Babikian et al. have innovated different films for wound care which can record electrical signals in a wound [35,36]. Both devices aimed to capture a wide range of physiological parameters using impedance, current, and other parameters.

**Figure 2:**
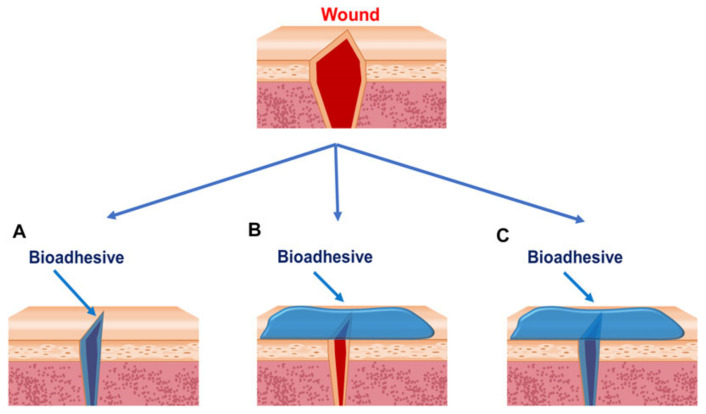
Strategies for bioadhesives used to close wounds. (**A**) Bioadhesives are applied between wound edges. (**B**) Bioadhesives are applied outside of the wounds. (**C**) Bioadhesives are applied between and outside the wounds. Reprinted with permission from Ref. [37].

Another dressing that aids wound healing is bioadhesives. These adhere to the site of a wound to moisten, absorb exudate, and protect from external pathogens [37]. These can be injected as hydrogels which then adhere to the surface of a wound or be delivered as a patch placed similarly to a plaster [38]. Figure 2 shows how a bioadhesive can cover a wound bed. Bioadhesives must be biocompatible and biodegradable; thus, they are often derived from nature. One bioadhesive formulated by Ke et al. used two natural products in tannic acid and silk fibroin to help develop the ECM and protect against bacterial infection [39]. The full extent of advanced dressings used for wound care is beyond the scope of this review, but it is clearly a growing field expanding into dressings that are responsive or indicative of physiological parameters. Table 1 summarizes the many kinds of wound dressing. Many of these dressings are being made using natural compounds because of their biocompatibility and biodegradability; thus, it is a promising field for natural products in wound care [40].

## 4. Natural Products

### 4.1. Modulators of Cellular Activity 

The third stage of wound healing, proliferation, can be hastened by compounds promoting cellular growth and proliferation. While its uncommon for this stage to be stalled or force the development of a chronic wound, increasing the proliferation rate can heal a wound faster to remove physical and cosmetic discomfort to the patient [18,20]. Increasing cell proliferation also means a lesser likelihood of scarring—increased proliferation in fibroblasts mainly corresponds with increased collagen and, therefore, a better-developed wound bed [46]. Table 2 shows the natural products discussed in this review’s mechanism of action for wound healing.

Curcumin, an extract from turmeric, is known for its bioactivity as an antioxidant, anti-inflammatory agent, antibacterial, and as a promotor of collagen synthesis and cell proliferation [46,47,48,49]. For these reasons, curcumin has been used by many cultures as a wound-healing drug applied topically to a wound [50]. Curcumin’s efficacy is limited by poor solubility in water and photosensitivity [46], hence its status as a traditional rather than conventional medicine [46]. 

Honey is another widespread medicine used traditionally for wound healing. The bioactive components of honey are a mixture of enzymes, pollen, and environment-specific molecules. However, some of honey’s more basic characteristics act to modulate cellular activity to promote wound healing [51]. Honey’s sugar content can provide nutrition to the cells fatigued from mass proliferation and inflammation to promote their survival and growth [52]. Honey’s low pH has also been reported to create conditions ideal for fibroblasts activity, facilitating better collagen development and fibroblast migration to close the wound [52]. Studies by Ranzato et al. further tested honey’s properties in an in vitro scratch wound healing model. They found honey to promote re-epithelisation and help close a wound in the final stages of its healing [53]. 

Another compound that promotes fibroblast proliferation and migration is tannin [54]. Tannin is a polyphenol that can be extracted from a myriad of plants and was traditionally used for tanning leather [55]. Alongside this purpose, plants such as *Entada phaseoloides* with high levels of tannins were used by South-East Asian cultures as a compound for wound healing. Su et al. studied the mechanism of action of tannin and found that it promotes fibroblast proliferation and migration. Increased cellular proliferation and migration quickens the wound-healing process and reduces the likelihood of scarring [54]. In this study, tannin acted similarly to the clinically used Bactroban with no significant difference between tannin from the *Entada* extract and Bactroban as a positive control [54]. Compounds such as honey, curcumin, and tannin used as traditional medicines have been analysed under modern pharmaceutical conventions to discover their modulation of cellular activity. A better understanding of this can allow future research to formulate such compounds into effective medicines [55,56].

### 4.2. Modulators of Collagen Synthesis

Synthesis of collagen returns structure and strength to the dermis after a wound [57]. Promoting collagen synthesis speeds up wound healing dramatically and reduces the risk of opening the wound again [20]. As well as the rate of collagen synthesis, the type of collagen being synthesised is important to promote non-scarred wound beds [58]. The importance of collagen in the wound-healing process has meant compounds that modulate the molecular mechanisms of collagen synthesis have been identified as wound-healing medicines.

Aloe vera is a natural compound still widely used and recognized in home, and clinical practices as a wound healing tool [59]. The most studied active ingredients of aloe vera are thought to be aloe-emodin, aloin, aloesin, emodin, and acemann [60]. While primarily known for reducing pain in burn wounds, aloe vera also increases the amount of collagen in wounds [61]. Beyond this, aloe vera affects collagen composition by promoting cross-linking; this develops greater three-dimensional structural integrity of the early granulation tissue and begins the return of the tissue to the regular dermis [62]. Aloe vera’s common form is a gel that not only delivers the drug but moistens the wound to improve flexibility, acts as a barrier against foreign microbes, and bathes the nerves to reduce pain [63]. 

Alkaloids are a class of basic, natural compounds, which contain at least one nitrogen atom. Alkaloids are present in many plants, particularly in flowers such as nightshade, poppies, and buttercups [64]. A study by Fetse et al. on alkaloids from *A. boonei* showed an increase in wound healing which was attributed to the promotion of re-epithelisation, angiogenesis, and increased collagen deposition [65]. In the same paper, *A. boonei* extract was theorised to be involved in collagen maturation: early granulation tissue contains collagen type IV while fully healed tissue contains the stronger and more developed collagen type I [65]. This paper found alkaloids to contribute to many different stages and components of the complex wound-healing process.

Collagen synthesis in cultured fibroblasts is increased by asiatic acid. Asiatic acid comes from the popular medicinal plant *Centella asiatica*, which has a long history in sub-continental traditional medicine [66]. There are three bioactive compounds in *Centella*: madecassoside, asiatic acid, and asiaticoside. All three were tested by Bonte et al. and then Maquart et al., who found each compound had positive effects on collagen synthesis in vivo. Asiatic acid, however, showed the most significant efficacy in vitro [67,68]. *Centella* extract contains all three extracts; however, Nagoor-Meeran et al. identified asiatic acid as the best compound to isolate for pharmaceutical use [66]. All three compounds have other properties ranging from antioxidant, anti-inflammatory, or anti-cancerous. *Centella*’s widespread historical use is clearly justified and only requires improved solubility and stability in the biological environment [3,4,69,70].

### 4.3. Modulators of Angiogenesis

There is a wide range of natural products which modulate angiogenesis. Both anti-angiogenic and angiogenic compounds can be used at different stages of the wound-healing process. Angiogenesis is a natural part of the proliferation phase of acute wound healing but can become problematic in a chronically inflamed wound [71]. Plant extracts to be used in both applications are common and widely used.

Plants containing flavonoids act as anti-angiogenic agents. Flavonoids are phenolic compounds with a benzo-γ-prone structure [72]. These compounds are found widely in tea, cocoa, and red wine, of which tea, especially, has been used as a natural wound-healing medicine [73]. Green tea contains a significant amount of flavonoids and other phenol-based compounds, which alter mi-RNA expression to restrict angiogenesis controlled by the VEGF receptor family [74]. Green tea in its traditional oral form is not specific to a wound, and as a topical extract, it cannot penetrate the stratum corneum [74]. Without specificity, green tea is limited despite promising bioactive properties. Despite this, green tea is a common anti-angiogenic solution, as well as other options such as *Centella asiatica* [74,75]. 

Aloe vera is a widely applied natural medicine that contains the pro-angiogenic compound beta-sitosterol [76]. Aloe vera’s pro-angiogenic activity makes it a candidate for promoting faster vessel growth and faster recovery in healing tissue. Beta-sitosterol works by stimulating vessel cell migration and enhancing the expression of angiogenesis-related proteins [76]. Aloe vera is widely available across the globe and is already used for other wound-healing properties, especially in burn wounds [59]. Aloe vera’s high water content can reduce wound-related pain while stimulating collagen synthesis and cross-linking directly hastens a wound’s natural healing process [63]. This, along with its pro-angiogenic properties, has made it a popular wound-healing drug across the world’s traditional medicine and continues to be one of the most widely recognised natural wound-healing solutions [59].

### 4.4. Modulators of the Extracellular Matrix

The ECM is a complex structure in the dermis that contains countless proteins, enzymes, and inflammatory components influencing how the body reacts to a wound [58]. There are fewer ECM modulating compounds than the other categories described in this review; however, their efficacy and common usage justify their acknowledgement as a natural product modulating the wound-healing process.

Stingless bee honey is one of the few natural ECM modulators. Honey was traditionally applied on wounds primarily because of its antibacterial and hydrating qualities; however, a study by Malik et al. has uncovered honey’s ECM modulating characteristics [57]. The unique mixture of enzymes, pollen, and other molecules in this honey down-regulated the MMP-1 gene, which encodes a protease protein, and up-regulated the gene for type I collagen, COL1A1 [57]. Reduced MMP activity and increased collagen will allow the development of collagen and extracellular proteins to recover the ECM earlier [77]. Malik et al. carried out this study in incubated human dermal fibroblasts and quantified gene expression using qRT-PCR [57]. Without an in vivo test, honey’s use as a wound-healing drug cannot be confirmed; however, Malik et al.’s findings add further potential to a new formulation of honey that can regulate fibroblasts in direct contact beneath the epidermis. 

Another compound belonging to bee honey, propolis, alters the expression and modification of ECM genes [78]. A notable protein altered by propolis is dermatan/heparan sulphate, a glycosaminoglycan which attracts water and cations to form most of the ECM’s content [79]. Its induction and structural modification, allowing growth protein attachment by propolis, increases the amount of the glycosaminoglycan in the dermis to recover the ECM quickly [79,80]. Propolis was compared to the widely commercially used silver sulfadiazine, which out-performed it in its induction of key ECM proteins such as dermatan sulphate and hyaluronic acid [81]. This outperformance of the ‘gold standard’ for ECM modulation presents propolis as a promising natural medicine that demands further research.

### 4.5. Modulators of Cytokines and Growth Factors

Cytokines are proteins or glycoproteins which modulate inflammatory or immune responses [95]. Growth factors increase cell proliferation, growth, and differentiation. Both classes of compounds modulate the progression of the wound-healing stages and the inflammation cascade [96]. Changing the timing or levels of these important compounds can change the balance of the cocktail of compounds controlling the body’s response to a wound. This critical role has meant a group of traditional medicines that alter the balance of cytokines and growth factors used specifically for wound healing [97].

Terpenoids are commonly found in essential oils and suppress cytokines to act as anti-inflammatories [82]. Marques et al. showed that terpenes reduced the pro-inflammatory cytokines TNF-α and IL-1α and increased production of IL-10. This had the net effect of suppressed inflammation to demonstrate its therapeutic potential [83]. In a systematic review, Barreto et al. found five preclinical terpenoids that showed potential as wound-healing drugs but were not well studied. Specifically, the mechanism of action requires better understanding to explain the clear evidence of anti-inflammatory activity [82]. The popularity of terpene products has risen with essential oils despite this poorly understood mechanism of action. The *B. morolensis* essential oil from Mexican folk medicine is an example of a terpene-containing essential oil that has been shown to promote wound healing [84].

Honey is a compound with many properties applicable to wound healing. One property is an effect on interleukins and MMPs. MMPs modulate the cleavage of cytokines controlling the inflammatory cascade and are a target enzyme to modulate cytokine levels [85]. Acacia and buckwheat honey especially showed this effect, while manuka, a more commonly known medicinal honey, showed a more minor modulatory effect [53,86]. Martinotti and Ranzato have also proposed a honey-based scaffold that can house growth factors to promote re-epithelisation [53]. This novel delivery system with honey, a known antibacterial and anti-inflammatory compound housing endogenous and modern synthetic growth factors, is a creative way to apply natural compounds to wound-healing medicine [52,86]. 

While not yet applied with natural compounds, cytokine modulators have been identified as novel therapeutic agents inhibiting fibrogenesis. Excess fibrogenesis often causes scarring and fibrosis of the skin; thus, modern cytokine modulators have become an active area of research [96]. Applying the modulatory effects of natural products such as terpenes and honey should be considered as another approach to prevent fibrosis. MMP modulators such as buckwheat and acacia honey should especially be considered as compounds preventing scarring and promoting re-epithelisation [53].

### 4.6. Natural Products Acting as Antibiotics or Antimicrobials

Antibiotic agents are important for the prevention and removal of bacteria from wounds. Bacterial invasion into a wound should be prevented at all measures as infection can form a chronic wound or develop into its own condition, such as gangrene or abscess [98]. Bacterial infections can become more complicated and severe than the wound itself hence the heavy emphasis in healthcare on antibiotic treatment of open wounds [77]. Products such as garlic, honey, and ginger have been used by ancient cultures as antibiotics to dress wounds and other bacterially inflicted conditions [5]. In the modern age of medicine, more efficient antibiotics such as penicillin have been developed; however, natural antibiotics continue to be commonly used worldwide [7].

Interestingly, many modern antibiotics are found in nature. Modern techniques have isolated active compounds from traditional antibiotics or antibiotics originating from fungi to achieve more refined and efficient use as pharmaceutics. An example of a traditional antibiotic is garlic. The compound responsible for its antibacterial activity is generally recognized as allicin, an organosulfur compound known to penetrate through the membranes of bacteria and interfere with essential enzymes [99]. Lu et al. investigated this in 2011 and had conclusive evidence of garlic concentrate’s efficacy against bacterial growth [87]. Garlic has been described as a medicinal plant in literature as far back as 6 BC and was used by ancient Sumerian, Egyptian, Indian, Chinese, and Greek healers [100]. Garlic is applied as a traditional ‘fix-all’ medicine, it is used as an anti-inflammatory agent, an antibiotic, or an anti-tumour product, amongst others [88,99]. While traditional medicines’ active compounds can inform the synthesis of novel antibiotic analogues, they should also be considered in modern applications, whether delivered traditionally or using modern pharmaceutical strategies. Petrovska et al. noted the value of garlic as an antibiotic even in the presence of the contemporary antibiotic, especially in an aged form [100]. Compounds trusted by many cultures such as garlic that have efficacy as antibiotics should continue to be researched to expand their natural potential.

Antimicrobials have been commercialised as an everyday supplement in the form of essential oils marketed as natural products curing all sorts of ailments ranging from anxiety to eczema [89,101]. A popular antimicrobial essential oil is lavender extract. Linalool, a chemical component of lavender, possesses antimicrobial activity [102]. Although essential oils are often designated as alternative medicine, the need for novel antimicrobial agents has directed research towards their efficacy [103]. Lavender oil has contradicting evidence for its efficacy; linalool was thought to increase bacterial cell wall permeability to increase another antimicrobial’s efficacy [104]. Guo et al. further studied linalool’s mechanism of antimicrobial action, finding that it not only ruptured cell walls but directly interacted with amino acid metabolism [90]. Other research has shown that linalool has little antimicrobial efficacy when used independently [105]. Predoi et al. found that linalool is not likely to prevent or improve microbial infections, contradicting Guo et al.’s growth curves showing linalool’s anti-microbial activity [90,105]. Essential oils such as lavender remind us that while traditional medicine is used for a reason, modern pharmaceutical analysis is required to determine a compound’s mechanism of action to formulate an effective delivery approach. Lavender has shown potential as an antimicrobial agent demanding further research and understanding to develop it into an effective therapy. 

### 4.7. Modulators of the Oxidant–Antioxidant Balance of the Wound Microenvironment

Antioxidants have shown promise in their ability to hasten the wound-healing process [86]. Antioxidants work by reacting with highly reactive radical oxygen species (ROS) to stabilise the ROS without becoming reactive themselves [106]. Aerobic metabolism in the mitochondria creates ROS proportionally to the metabolic demands on the cells. The increased proliferation and migration occurring during wound healing elevate metabolic demand and ROS presence [86]. ROS serves essential functions in phagocytic and cell-signalling mechanisms but can also cause oxidative stress at high levels; therefore, their presence is tightly regulated [86]. Oxidative stress can degrade membranes, DNA, lipids, and protein in the skin, killing fibroblasts and tightening skin [107]. ROS and inflammation are tightly linked as they are elevated during inflammation but can also induce the process [46]. This forms a positive feedback loop and can contribute to a wound becoming chronically inflamed [108]. Antioxidants are released endogenously by the body’s ROS regulatory mechanism but are also a popular diet supplement present in vegetables, berries, and other food groups [109]. Applying antioxidants systemically or locally can help balance ROS levels to reduce oxidative stress and enhance wound healing [106].

Curcumin has been used as an antioxidant in traditional medicine and is an example of a natural antioxidant requiring advanced delivery techniques. The active ingredient in turmeric, curcumin, possesses anti-inflammatory, anti-infective, and antioxidant properties [47]. Curcumin’s use is limited by its low water solubility and stability, making effective delivery to the predominantly aqueous wound environment difficult [47,56,110]. Gopinath et al. proved curcumin’s antioxidant ability by demonstrating a decrease in curcumin and the ROS oleic acid when combined using time-dependent studies. Results showed reduced absorption corresponding to decreased curcumin, indicating reactions between oleic acid and curcumin [41]. Curcumin’s antioxidant activity was confirmed by analysing the expression of an endogenous antioxidant enzyme: superoxide dismutase (SoD). Curcumin’s efficacy in stabilising ROS levels reduced the need for endogenous antioxidants; therefore, SoD’s expression was reduced compared to control [41]. In multiple trials, Panahi et al. have also shown the effectiveness of curcumin as an antioxidant in humans using a dosage regime and blood testing [47]. On top of these experiments proving curcumin’s antioxidant ability, Gopinath et al. and Zhao et al. have demonstrated curcumin’s efficacy as a wound-healing drug [41,56]. These trials used a composite dressing and a collagen film, respectively, to improve the bioavailability of the drug and showed significant wound-healing ability of curcumin-loaded dressings/films [41,56]. Curcumin is an example of a traditional compound used by many different cultures which have antioxidant properties with the potential for better delivery [50].

Vanillin, a phenolic aldehyde found in olive oil and vanilla pods, is an antioxidant and an antimicrobial compound. This natural compound modifies oxidant balance by reacting with radicals in a self-dimerisation reaction to clear the wound bed of ROS [91]. When compared to ascorbic acid, vanillin showed superior anti-oxidising ability in ABTS(+), ORAC, and OxHLIA assays but no activity in the DPPH or galvinoxyl assays [91]. When applied in a dressing or hydrogel, vanillin has been shown to prevent ROS proliferation and bacterial infection in wounds [92,93]. Vanillin’s bioactivity is a recent discovery; thus, it is a popular area of research.

### 4.8. Other Natural Products with Wound-Healing Properties

Although rarely seen as a treatment, monitoring intake of vitamins A, B, C, and D can impact the rate and extent to which a wound heals [111,112]. These vitamins are involved in collagen synthesis and inflammation and a deficit in vitamin levels during wound healing can lead to chronic wound development. Vitamin C is particularly well known as a common vitamin involved in wound healing which can cause scurvy if at low systemic levels [113]. Another notable compound is vitamin A and other associated retinoids. The efficacy of local retinoids is debated, and the mechanism of action is not fully understood [114]. Despite this, they are commonly used in dermatology as a topical ointment. Vitamin A is a compound whose delivery could be improved. Systemically, it is a detriment to wound healing, meaning delivery must be highly localised and separate from systemic circulation [94]. Vitamins are notable traditional medicines that are extensively researched and are at the forefront of many modern delivery adaptations [115]. 

There are countless herbs and plant extracts used in the many different cultures worldwide as wound-healing medicines. A more comprehensive list was compiled by Agyare et al., which lists many compounds less studied and not discussed in this review [97].

## 5. Advanced Delivery Strategies for Natural Wound-Healing Compounds

**Figure 3:**
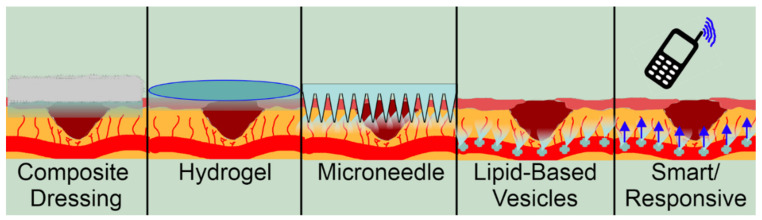
Modern delivery methods applied to natural products to improve their delivery and efficacy.

As our understanding of drugs have advanced, so has our ability to deliver them. As important as discovering novel drugs is discovering novel ways to deliver them. Older technologies such as dressings have been transformed into composite dressings which deliver a drug while still protecting the wound from the outside environment. This combination of drug and dressing adds functionality while maintaining the original purpose of a dressing. Similarly, hydrogels able to deliver a drug, moisten a wound, and protect it from the outside environment can remove the need for multiple wound treatments. Hydrogels are formed from water and polymers cross linked to form a gel with pores able to store a drug. Other techniques are the product of innovation, such as microneedles, lipid-based vesicles, and smart/responsive delivery. Microneedles are usually patches with tiny needles extending from their surface designed to penetrate the stratum corneum to access the dermis. Microneedles are painless and self-applicable and therefore are favoured over syringe-based applications. Lipid-based vesicles house drugs inside a bilayered sphere for transport through lipophilic membranes. These are often used for systemically applied drugs. Finally, smart/responsive delivery methods use environmental cues or remote control to release a drug at specific times and locations. This can be applied to most techniques and is able to release a drug at a specific moment in the temporally complex course of wound healing. Figure 3 graphically shows the different strategies and how they store and release a drug to the dermis.

### 5.1. Composite Dressings

Composite dressings use the traditional dressing format but include a drug in the dressing itself. Dressings aim to allow oxygen exchange, maintain moisture, and protect the injury from infection [116]. Natural products which are biodegradable, versatile, and sustainable can be applied as biopolymer or biocomposite dressings. These can be more than just a dressing by promoting ECM regrowth, preventing scarring, or influencing inflammation [117]. Biodegradable dressings which produce no medical waste are of increasing interest as emissions and pollution are increasingly scrutinised [118]. 

A growing field is the delivery of growth factors by composite dressings. Modulating growth factors can speed up wound healing and promote an acute wound instead of a chronic wound [13]. Yao et al. developed a collagen sponge as a composite dressing to deliver endogenous growth factors to chronic ulcers and tested it in double-blinded controlled trial [119]. Their results showed an increased incidence of complete wound closure, a shortened healing time, and improved healing quality [119]. Using a dressing that delivers an endogenous therapeutic substance decreases the chance of an immune reaction or instability. Catanzano et al. discussed the potential of such systems, concluding that they will soon reach more widespread clinical use [120].

Composite dressings can contain natural products for delivery or be composed of a natural product itself [121]. An example of such a dressing was created by Gao et al., who used chitosan and diazo resin to create a hydrogel composite dressing [27]. Chitosan and diazo are both sourced from natural sources and are known antibacterial agents. Together with their antibacterial activity, the dressing showed an increased rate of wound healing [27]. Chitosan can be prone to lysosomal attack; thus, other combinations have been included with chitosan to improve composite dressings’ efficacy. An alginate/ZnO_2_ composite bandage formulated by Mohandas et al. showed desirable antibacterial effects against methicillin-resistant staphylococcus aureus and increased keratinocyte migration towards a wound area [122]. This formulation also included a chitosan hydrogel that worked with the ZnO_2_ and alginate to achieve a therapeutic effect with less toxicity than a purely chitosan-derived gel [122]. The composite dressings formulated by Mohandas et al. and Gao et al. improve a traditional wound-healing structure; however, some researchers are innovating new forms of natural-product-based dressings altogether [123]. 

Electrospun nanofiber mats effectively deliver drugs topically while also acting as a wound dressing [124]. Many traditional wound-healing compounds such as curcumin from turmeric or thymol from thyme are lipophilic and are therefore challenging to localise when treating [125]. Nanofiber mats are created by charging droplets of a polymer and slowly releasing it across an electrical field to form a mat or sheet made up of long strands of tiny diameter—this creates space between fibers where a drug can be encapsulated [42,124]. Formulating a lipophilic drug this way allows the drug to be administered directly to the wound while the mat acts as a moistening dressing. Research reported that essential oil-loaded nanofiber mats showed more effective anti-inflammation/healing/antimicrobial activity (depending on the compound used) compared to essential oil itself [124,125]. Thymol was effective using this method—thymol’s lipophilicity meant it could leave the mat and diffuse towards the wound quickly. In Garcia et al.’s study, thymol delivered by nanofiber mats showed similar anti-inflammatory properties to the commonly used drug dexamethasone, suggesting thymol as an effective natural alternative [125]. Electrospun nanofibrous structures could increase traditional medicine’s efficacy, which was previously difficult to deliver due to their high lipophilicity locally.

Foams for wound healing are typically formed from synthetic polymers used as a replacement for gauze. Foams can be used to deliver drugs, moisturise wound beds, or manage exudate [126]. The release profile of foam dressings is typically rapid due to their free form; however, they usually require another bandage/dressing to prevent infection [33]. One such foam dressing was designed by Bai et al., which delivered a mix of silk fibroin protein, gastrodia elata, and tea tree oil [43]. Their foam dressing showed accelerated wound recovery by generating more abundant and thicker collagen in the dermis [43]. Foam dressings and other composite dressings are common ways of developing natural products historically and are now an area of active research. Technologies such as electrospun nano mats improve how natural products can be delivered while retaining the known benefits of dressing a wound.

### 5.2. Hydrogels

Hydrogels form a moistening barrier to the outside world at the surface of a wound to protect from foreign microbes while keeping the wound moist [125]. Therapeutic compounds are stored in the pores of hydrogels to be released onto the wound. When forming a hydrogel, a polymer with correctly sized pores is required to maximise drug release from the gel to the wound. Hydrogels delivering curcumin, aloe vera, and cordycepin have been designed successfully as novel delivery strategies [28,49,127,128]. Song et al.’s development of a cordycepin/chitosan complex hydrogel is an excellent example of a natural-product-based gel. This hydrogel showed desirable structural, swelling, and mechanical properties as well as a self-healing ability. The ability to self-heal allows the gel to mould itself to the status of the wound rather than require pre-moulding. Song et al. showed improved re-epithelisation and increased collagen deposition in wounds with the gel compared to a control [28].

More complex hydrogels such as curcumin/chitosan nanoemulsions have also been developed. The antibacterial chitosan stabilises curcumin in a hydrogel to create an antibacterial dressing that moistens the wound while delivering curcumin as an antioxidant [49]. Aloe vera has also been formulated into a nanohydrogel that polarises macrophages to modulate their activity during wound healing [128]. Nanohydrogels have much smaller pores than regular hydrogels, which allows ‘smart’ hydrogel development to alter their properties in response to external stimuli [128]. The nanohydrogel (made by aloe and chitosan) reported by Ashouri had shown significant changes in cytokines involved with macrophage polarisation when the cells became closer to an M1 subtype [128]. Future treatments responsive to changes in the wound environment can be designed to change their output to direct wounds away from becoming chronic and reduce the need for constant changing of dose and drugs [2].

Hydrogels not only deliver natural compounds but can also be fabricated from naturally occurring polymers. These polymers can be safer and more biocompatible than other synthetic polymers [26]. Zhi et al. created natural-product gels composed of self-assembling triterpenoids [129]. The triterpenoids were taken from Chinese medicinal herbs and used as a gel scaffold to deliver DOX-1 to treat murine tumours [129]. The gels showed an excellent network polymer structure and a sustained release profile comparable to other synthetic compounds [129]. Triterpenoids have antioxidant activity, which can complement the anti-tumour activity of DOX-1. Rather than giving both compounds in tandem, this study pioneered a system that could deliver DOX-1 with a triterpenoid-based structure. Another natural gel uses a decellularised ECM to create a hydrogel that replicates the (ECM when delivering a drug [44]. This helps the ECM return quickly while causing the least disruption possible to a wound. This hydrogel application promotes tissue remodelling and cell proliferation and can deliver a select drug to the application site. These novel hydrogel formulations show how hydrogels can deliver natural products and form the scaffold of the hydrogel.

Hydrogels allow drugs to be delivered locally to a wound without the need for a clinician. Combined with their ability to act as a dressing, hydrogels have become a popular prescription for clinicians and an emerging field for delivering traditional compounds [127,130].

### 5.3. Microneedles

With the turn of the century, microneedles have emerged as an exciting new method for transdermal drug delivery [2]. The traditional transdermal or subcutaneous delivery method of hypodermic needle injection results in medical waste, requires a clinician, and is traumatic. Microneedle technology is a relatively painless and frequently self-administered approach for drug delivery. Drugs with wound-healing ability are often formulated and tested in the microneedle format [131]. There are five classes of microneedles: solid, hollow, coated, dissolving, and hydrogel-forming [2]. Solid microneedles do not have drugs loaded onto the microneedles but disrupt the skin to create pores for a topically applied drug to enter the following microneedling. Hollow microneedles contain an internal pore that can hold a larger volume of drug to be released into the dermis [132]. Coated microneedles have a similar makeup to solid microneedles but are coated in the drug before application. Dissolving and hydrogel-forming microneedles differ from solid/coated microneedles by being left in the skin for an extended period. Dissolving microneedles have the drug incorporated into their own needles. When inserted into the dermis, the needles dissolve to release the drug. Hydrogel-forming microneedles can have the drug in the needles or in the patch supporting the microneedles and work by partially dissolving to create a liquid channel between the drug-loaded patch and the needles into the dermis [132]. As the field has advanced, so has the design of microneedles. ‘Smart’ microneedles can modulate their release of anti-inflammatory compounds according to inflammation-induced temperature changes [133]. Smart microneedles can reduce the need for clinical care of an inflamed wound and prevent the formation of a chronic wound in an inflammation loop [108]. Park and Frydman reported that the antibacterial, antioxidant, and wound-healing effects were significantly increased using microneedles loaded with manuka honey and green tea extracts, compared to traditional formulations such as cream or topical solutions [51,134]. 

Microneedles are not just used to bypass the stratum corneum but can also pierce the bacterial biofilm often present in chronic wounds. Biofilms are impermeable to most drugs and can be difficult to remove. The development of microneedles as an alternative to the painful and dangerous hypodermic needle has also introduced it as a new delivery strategy for the removal of biofilms [1,2,132]. Frydman and Chi both reported that the antibacterial-agent-loaded microneedle arrays could effectively remove or inhibit the growth of biofilms [51,133]. Biofilms are present in conditions other than wound healing and demand constant vigilance from healthcare workers to prevent their formation on medical equipment or open wounds. Antibacterial microneedle patches composed of manuka and chitosan have great potential to remove biofilms in wounds and other contexts, thus making antibacterial microneedles a hot topic in research [2].

### 5.4. Lipid-Based Vesicles/Nanotech

Molecules that are difficult to deliver can be encapsulated into lipid-based vesicles to improve their solubility and stability [135,136]. Most natural products are biologically unstable, insoluble in water, and poorly distributed to target sites. Lipid-based vesicles can improve their solubility and stability to improve their delivery [137]. 

Liposomes form double-chain phospholipids to form a bilayered sphere with a hydrophobic layer surrounded by a hydrophilic core and surface [135,136,138]. Compounds such as thymol and carvacrol from oregano which have poor solubility and stability can be encapsulated in liposomes to improve their bioavailability and delivery. Liolios et al. reported that both thyme and carvacrol showed better distribution and stability, resulting in greater efficacy [136]. Liolios et al. were not targeting wound healing with their study; however, others have used liposomes in this context. Given that thymol and carvacrol have proven efficacy in wound healing as antioxidants and antimicrobial agents, there is an opportunity to develop a liposomal formulation of the drugs aimed at wound healing [139]. Li et al. used madecassoside from *Centella asiatica* in biodegradable liposomes targeted at burn wounds in rats [140]. This formulation improved madecassoside’s delivery by enhancing its stability and controlling its release. The significant findings in Li et al.’s study demand further research into the many other lipophilic natural compounds. Liposomes are useful vehicles for typically insoluble drugs and more studies on their efficacy in wound healing will hopefully be carried out.

Another lipid-based vesicle, which drugs can be encapsulated in, is a niosome. Niosomes are also bilayered spheres but differ from liposomes being formed from non-ionic single-chain surfactants and amphipathic compounds [135,141]. Niosomes are easier to fabricate and are more stable than liposomes. Niosomes are less expensive and do not require unique methods for handling and purification [135]. These favourable characteristics have brought niosomes to the forefront of lipid-based vesicle research—many consider niosomes to be the future of the field with widespread applications across medicine and nutrition [135,142]. Curcumin’s solubility, stability, and efficacy against cancer cells were improved in a niosomal formulation compared to a dissolved curcumin solution [143]. Similarly to Akbarzadeh et al.’s study, Xu et al. applied a niosomal formulation to the long process of wound healing [144]. Xu et al.’s curcumin-loaded niosomal formulation displayed a controlled release profile reaching 80% release after 25 h and a significantly greater cellular uptake compared to free curcumin dissolved in solution [144]. Such characteristics and promising results suggest that niosomal formulation could be ideal for wound healing. 

Exosomes are another lipid-bilayer spherical vesicle that can carry both hydrophilic and hydrophobic drugs. Exosomes differ from liposomes and niosomes in the complexity of their surface, which contains organotrophic proteins allowing directed transport inside and outside the cell [145,146]. Laborious production, safety issues, and low yield have slowed the development of exosomes, with only natural exosomes being viable to form [145]. These natural exosomes from fruit and dairy have not shown much advantage over liposomes other than increased safety and biocompatibility [147]. Sun et al. formulated curcumin into exosomes to protect the drug from biodegradation (over 2.5 h) and only a quarter amount of curcumin was degraded compared to curcumin dissolved in solution [148]. This paper focused on curcumin’s anti-inflammatory activity, which can be easily applied to a wound-healing context. Some reviewers of lipid-based vesicles encourage mixing liposomes, exosomes, and niosomes to use together [137,145]. A suggested solution is to engineer the organotrophic proteins of exosomes onto niosomes or liposomes to create targeted easily produced vesicles. While lipid-based vesicles have been used in wound-healing applications to deliver natural compounds, only a few studies describe how natural compounds can be fabricated into vesicles and be delivered to wounds. 

Nanocarriers can be used to control or optimise the delivery of drugs. As with microneedles and hydrogels, nanocarriers can be designed to be responsive to external stimuli, making them smart nanocarriers [149]. For drugs that struggle to bypass membranes or have undesirable pharmacokinetic characteristics, nanocarriers can help drugs diffuse across lipophilic membranes and optimise their release. Thymol extracted from oregano and thyme has been formulated into a chitosan/tragacanth gum nanocarrier by Sheorain [150]. Thyme was used by ancient Mediterranean cultures as an antioxidant and antimicrobial agent but was limited by low bioavailability and insolubility [151]. Sheorain’s study improved both characteristics by encapsulating thymol into a chitosan/tragacanth gum nanocarrier that has antibacterial properties derived from chitosan. As a result, thymol displayed more significant antioxidant activity (as measured by ROS-scavenging activity) at multiple doses formulated in nanocarriers rather than its natural form [150]. Using such carriers can harness the effectiveness of traditional compounds such as thymol which need a better formulation to be delivered effectively. 

Similarly to nanocarriers, porous microspheres encapsulate a drug and alter the drug’s absorption and release profile. Porous microspheres act more as reservoirs than nanocarriers—they protect the drug avoiding inactivation from the external environment and prolonging the drug’s absorption [152]. This technology was effectively applied to the traditional compound of asiaticoside by Zhang et al. [153]. Asiaticoside has a variety of favourable pharmacological characteristics as a wound-healing drug but is limited by its poor solubility and encapsulation inside a soluble microsphere successfully facilitated its entry into a wound and sustained its release over time to achieve a more favourable time/concentration relationship absorption [153]. 

### 5.5. Responsive and Smart Delivery

At the forefront of drug delivery innovation are smart/responsive delivery systems. A smart system can respond to endogenous or exogenous stimuli to alter drug release. These technologies aim to modulate drug release according to properties such as pH, glucose levels, and temperature as a gauge of a wound’s status [154]. Identifying how these properties reflect changes in the wound has been the rate-limiting step in this field, meaning few examples currently exist [45]. Such devices show great potential for delivering natural products in a sustained and controlled fashion. This field is rapidly expanding and will continue to develop systems applicable to a wide variety of wound-healing drugs [155].

Inadequate oxygenation is a known cause of wounds becoming chronic; therefore, the oxygen content is a property commonly sensed by smart delivery systems. One such system was developed by Ochoa et al. as a biocompatible patch that can deliver oxygen to a wound bed when oxygenation decreases too far [156]. While not yet developed, a similar patch that senses perfusion could deliver natural antioxidant products to prevent ROS production when perfusion is fluctuating. Compounds such as curcumin, which can be loaded into nanofibers with smart-delivery potential, should be investigated for this purpose [157]. 

Smart devices can also respond to exogenous stimuli such as smartphone activation. Tamayol et al. produced nanofibrous textile platform that could respond to such external stimuli to release chitosan-based nanoparticles [158]. The platform included flexible heaters responsive to an external signal that triggers thermosensitive nanoparticles to release into a wound. Mostafalu et al. used the same system to deliver VEGF and antibiotics in a controlled and sustained manner [159]. Their system demonstrated efficacy and controlled drug release profiles, further showing that smart delivery systems should continue to add a new dimension to drug delivery.

## 6. Future Perspectives

Growing distrust of pharmaceutical companies and governments has raised public and private interest in traditional medicines over the past few decades [160,161]. Studying these compounds and improving their delivery can lead to quickly commercialised products. Some markets are likely to be more receptive to a wound-healing drug synthesised from thyme than a drug developed in silico or from a library [162]. The current work being carried out to improve the delivery of traditional compounds can improve drug efficacy and add academic legitimacy to the use of natural products. 

85% of the world’s population rely on plant products for their medicine, mostly in developing countries that have less access to modern synthetic drugs [163,164]. Table 3 further shows a correlation between the use of traditional medicine in developing nations and geographic location and income. Oyebode et al. showed that people of lower socioeconomic status that are unemployed, live in rural areas, and report a lower health status are more likely to use traditional medicine [165]. Medicine development aimed at developing countries should consider natural products already established in that country or culture to maximise accessibility. While growing, there are few pharmaceutical labs in developing countries that are able to create and apply such treatments [166,167], which is unlikely to change and is a critique of this approach. Another problem is maintaining the allure of natural products when delivered non-traditionally—such an approach may remove the appeal of natural products. 

A potential concern is tarnishing a drug’s appeal as a natural product by using complex pharmaceutical delivery techniques. For example, traditional medicines such as curcumin or manuka honey may not be culturally accepted if loaded into nanocarriers. This field’s future is toeing the line of compounds that are accepted as natural while remaining effective in their delivery and method of action. Microneedles are a promising field with an increasing amount of research going into it. Microneedles formed of compounds such as chitosan or made from the active compound itself (such as Frydman’s manuka microneedles) have the potential to be marketed as totally naturally based [51]. Nanocarriers, nanofibers, hydrogels, and microneedles also improve their encapsulated drugs’ access to a wound while removing the need for systemic administration or subcutaneous injection [15]. All four of these technologies also have the potential to be ‘smart,’ where they can respond to stimuli such as ultrasound or temperature to change their physicochemical characteristics. Some of the most successful formulations of natural compounds have been vesicle-based smart delivery systems delivering catechin, thymol, curcumin, and madecassoside [138,168,169,170,171,172].

Modern nanotechnology has led to novel techniques such as 3D printing and lithography being applied to drug delivery. Lithography has been applied to microneedle formation and to survey endothelial cell migration in the field of wound healing [173,174]. The process of 3D printing using bio-inks has also been tested in the area with promising results: in vitro and in vivo animal studies of bio-ink engineering promote wound healing [175]. Indeed, the field of wound healing will keep expanding as technological advances do. 

## 7. Conclusions

It is important to apply advanced delivery systems to natural products used traditionally. This benefits developing countries where traditional medicines are more accessible. Many traditional medicines have been shown to be efficacious and should no longer be considered an inferior approach to therapy. The delivery of traditional medicines is one of the most common problems with these drugs; thus, modern delivery techniques should be applied to maximise their natural potential. The authors believe that advanced drug delivery systems would improve the therapeutic efficacy of traditional wound-healing medicines, placing natural compounds such as curcumin and thymol at the forefront of the wound-healing field.

## Figures and Tables

**Table 1:** Characteristics of wound dressings.

Type of Wound Dressing	Features	Limitations	Product Name	References
Film dressings	Elastic, durable, comfortable, and conform well to body contours Waterproof and transparent Create a moist healing environment Bacterial and viral barrier Semi-permeable to water vapour and gas	Adhesive films might disrupt newly formed epithelium during dressing change Limited use for highly exuding wounds Might develop leakage channels	Tegaderm™ (3M™, UK Plc.) Opsite Films^®^ (Smith and Nephew) Mepitel^®^Film (Mölnlycke Health Care Limited)	[32,34,41]
Foam dressings	Highly absorbent Easily removable Create a moist healing environment Bacterial and viral barrier Semi-permeable to water vapour and gas	Form an opaque layer, making wound monitoring difficult Limited use for dry wounds Poor stability	Flexsan Biopatch Biatain Cultinova Lyofoam Allevyn Unilene Tielle CuraSpon Kendall Hydrasorb	[33,42]
Hydrogel dressings	Create a moist healing environment Pain relief Self-applied or injectable Facilitate autolytic debridement Easily removed	Require a secondary dressing Lack of mechanical strength Inconsistent hydration properties Poor bacterial barrier	Suprasorb^®^ AquaDerm™ Neoheal^®^ Simpurity™ DermaGauze™ Restore	[43,44,45]
Bioadhesive dressings	Create a moist healing environment Self-injectable Adhesive	Unremovable Interference with other medical devices Might develop leakage channels	Ligate™	[37]

**Table 2:** Wound healing natural products are classified into their mechanism of action.

Mode of Action	Natural Products	References
Modulators of Cellular Activity	Turmeric, Honey, and *E. phaseoloides*	[46,47,48,49,50,51,52,53,54,55,56]
Modulators of Collagen Synthesis	Aloe vera, *A. boonei, C. asiatica*	[57,58,59,60,61,62,63,64,65,66,67,68,69,70]
Modulators of Angiogenesis	Honey, Aloe Vera, and *E. phaseoloides*	[59,63,71,72,73,74,75,76]
Modulators of the Extracellular Matrix	Honey	[57,77,78,79,80,81]
Modulators of Cytokines and Growth Factors	Essential Oils and Honey	[52,53,82,83,84,85,86]
Antibiotics and Antimicrobials	Garlic and Lavender	[87,88,89,90]
Modulators of Oxidant/Antioxidant Balance	Turmeric and Vanilla	[41,91,92,93]
Other	Vitamins A/B/C/D	[94]

**Table 3:** Adjusted odds ratios and 95% confidence intervals of users of traditional healers by demographics in China, Ghana, and India. Adapted with permission from Ref. [165]. Copyright 2016, Oyebode.

China		
	OR (CI)	*p*-Value
Rural	6.9 (5.4–8.9)	<0.001
Income quintile	1.2 (1.1–1.2)	<0.001
**Ghana**		
	**OR (CI)**	***p*-Value**
Rural	1.4 (1–2.2)	0.077
Income quintile	0.8 (0.7–0.9)	0.002
**India**		
	**OR (CI)**	***p*-Value**
Rural	1.3 (0.9–2)	0.217
Income quintile	0.8 (0.7–0.9)	0.001
